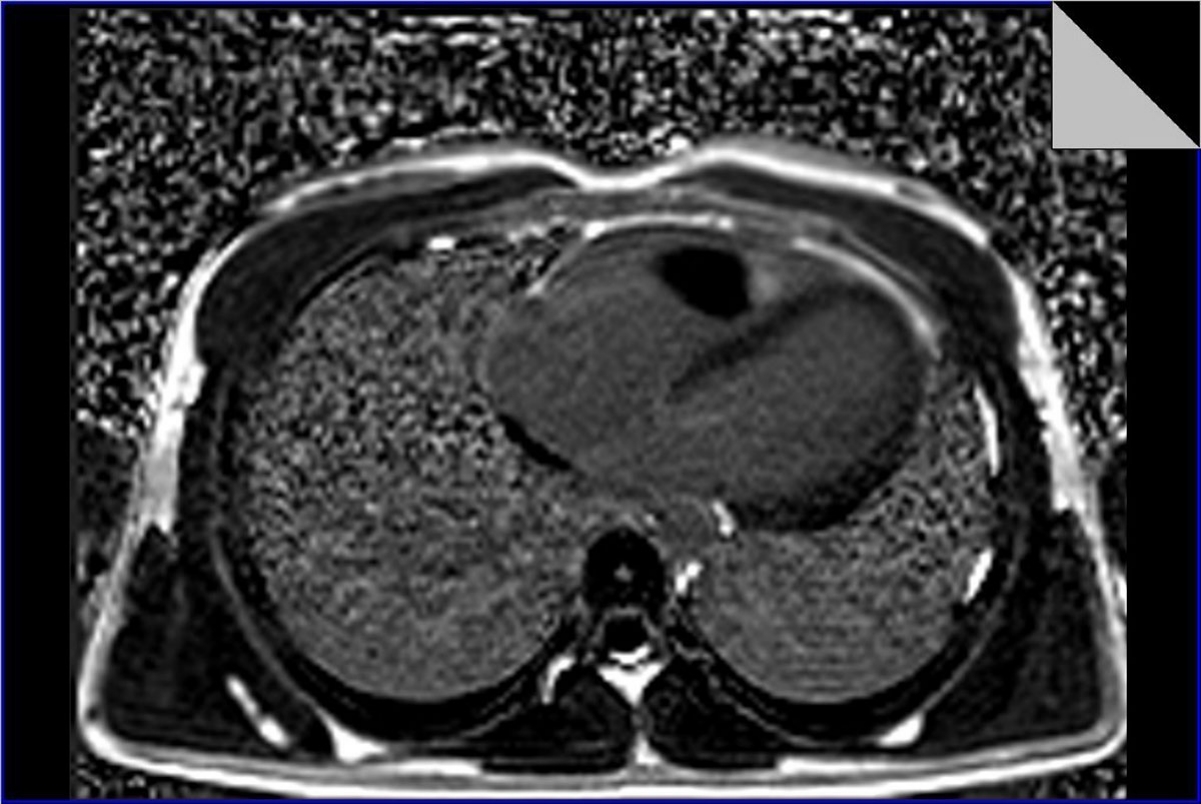# Report of a right ventricular outflow tract myxoma in a young adult

**DOI:** 10.1186/1532-429X-17-S1-P374

**Published:** 2015-02-03

**Authors:** Sara El Fawal, El Atafy El Metwally

**Affiliations:** 1 Radiology, Faculty of Medicine, Alexandria University, Alexandria, Egypt; 2 Cardiothoracic Surgery, Tanta University, Tanta, Egypt

## Background

Primary cardiac masses are rare and usually benign. Myxomas are usually seen in adults, accounting for 25%-40% of all cardiac tumors from birth to adolescence. The majority of myxomas (75%) are located in the left atrium.

*Right ventricular myxomas* are very rare, only found in 2%-4% of cases.

The most important distinguishing feature of a myxoma is the characteristic narrow stalk and tumor mobility, although sessile, non-mobile myxomas can also occur.

Myxomas may be homogeneous or may have central areas of heterogeneity representing hemorrhage and necrosis. Calcification may also be detected.

*In adults*, differential diagnosis for an intracavitary cardiac mass includes thrombus, myxoma, lipoma and nonmyxomatous neoplasms.

## Methods

1. Echocardiographic examination.

2. Cardiac magnetic resonance imaging examination.

3. Surgical resection of the mass.

4. Pathological examination of the resected mass.

## Results

1. *Echocardiography* revealed a very large elongated mass attached to the pulmonary valve extending into the right ventricular outflow tract.

It was seen protruding through the pulmonary valve causing partial obstruction, highly suspicious of infective endocarditic mass, for surgical consultation.

2. Cardiovascular magnetic resonance imaging examination:

Cine steady state free precession (SSFP) sequences revealed A large mass is seen arising from the right ventricular free wall close to the right ventricular apex with a relatively small pedicle and possible attachment to the moderator band.

It projects superiorly into the right ventricular outflow tract (RVOT), it is relatively mobile and protrudes through the pulmonary valve in systole with no obvious attachment. It measures about 8x3x5.5cm in max dimensions.

It is mostly isointense on black-blood imaging (T1 & T2WI), with small hyperitense foci.

No appreciable enhancement on perfusion imaging.

Myocardial delayed enhancement sequences showing non enhancing main bulk of the lesion, with a smaller enhancing portion at its anterior and inferior portion (site of suspected origin) with subtle patch of delayed enhancement in its most superior portion as well.

The right ventricle is mildly dilated with diminished global systolic function (ejection fraction=48.5%). The pulmonary arteries are of average size. The right atrium and inferior vena cava are relatively dilated.

Overall features are of a sizable right ventricular mass projecting into the RVOT, signal intensity and enhancement characteristics are suggestive of a myxoma.

3. *Operative report* showed the the mass was attached to the right ventricular free wall, with no attachment to the pulmonary valve, it was easily resected.

4. *Pathology report* described a cardiac myxoma.

## Conclusions

In case of a benign-looking, pedunculated, relatively mobile, rather heterogeneusly enhancing cardiac mass is encountered, the possibility of a myxoma should be included in the differential diagnosis, even if the mas is present in an unusul location like the right ventricle.

## Funding

Funding was provided by the Department of Radiology, Faculty of Mdicine, Alexandria University.

**Figure 1:**
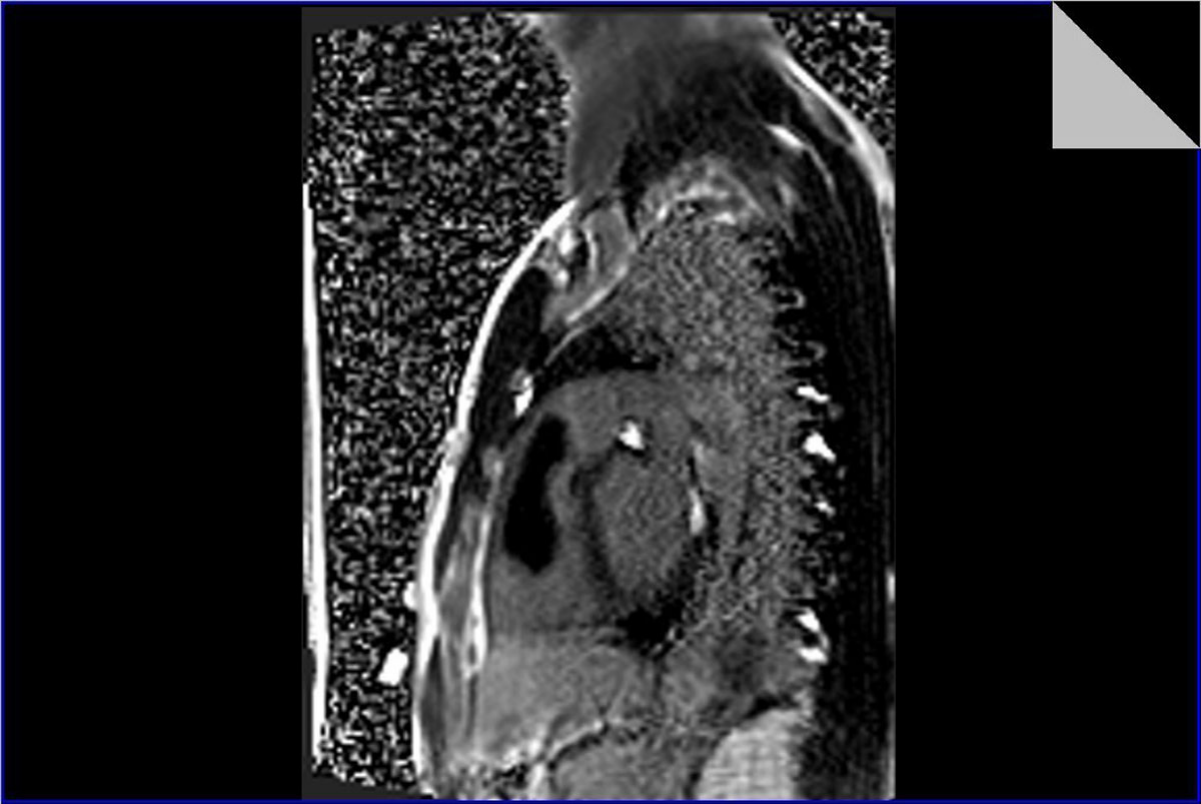


**Figure 2:**